# ACTIVITY INTENSITY AND FALL-RELATED DEATHS AMONG OLDER ADULTS: RESULTS FROM A 12-YEAR NATIONAL SURVEY

**DOI:** 10.1093/geroni/igae098.4355

**Published:** 2024-12-31

**Authors:** Oluwaseun Adeyemi, Joshua Chodosh

**Affiliations:** New York University Grossman School of Medicine, New York, New York, United States; New York University Grossman School of Medicine, New York, New York, United States

## Abstract

Fall-related deaths among older adults are a growing public health concern, with activity intensity potentially playing a critical role in mitigating these outcomes. This study assessed the relationship between activity intensity and fall-related death among community-dwelling U.S. older adults. We pooled twelve years of survey data (2006 to 2017) from the Integrated Public Use Microdata Series of the National Health Interview Survey for this retrospective cohort study. We identified older adults (65 years and older) who sustained fall injuries (N=2,454). The outcome variable was fall-related death. We defined low activity intensity as a binary variable using the American Heart Association’s weekly 500 Metabolic Equivalent of Task (MET) as a cut-off. We controlled for sociodemographic, healthcare access, and health characteristics, performed survey-weighted logistic regression analysis, and reported the adjusted mortality odds (plus 95% confidence interval (CI)). The survey comprised 2,454 older adults with fall injuries, representing 863,845 US older adults. The population was predominantly female (68%), non-Hispanic Whites (85%), and divorced/separated (54%). During the follow-up period, 45% of the study population died. Approximately 81% of the study population had low activity levels. However, between 2006 and 2017, the proportion of the study population with low physical activity decreased from 90% to 67%. After adjusting for sociodemographic, healthcare access, and health characteristics, low activity level was associated with two-fold increased odds of death (AOR: 2.11; 95% I: 1.59 – 2.81). Promoting higher physical activity levels may significantly reduce the risk of fall-related deaths among older adults.

